# Intrinsically Photosensitive Retinal Ganglion Cells (ipRGCs) Are Necessary for Light Entrainment of Peripheral Clocks

**DOI:** 10.1371/journal.pone.0168651

**Published:** 2016-12-16

**Authors:** Paulo Kofuji, Ludovic S. Mure, Logan J. Massman, Nicole Purrier, Satchidananda Panda, William C. Engeland

**Affiliations:** 1 Department of Neuroscience, University of Minnesota, Minneapolis, Minnesota, United States of America; 2 Regulatory Biology Laboratory, Salk Institute for Biological Studies, La Jolla, California, United States of America; Morehouse School of Medicine, UNITED STATES

## Abstract

Light is a powerful entrainer of circadian clocks in almost all eukaryotic organisms promoting synchronization of internal circadian rhythms with external environmental light-dark (LD) cycles. In mammals, the circadian system is organized in a hierarchical manner, in which a central pacemaker in the suprachiasmatic nucleus (SCN) synchronizes oscillators in peripheral tissues. Recent evidence demonstrates that photoentrainment of the SCN proceeds via signaling from a subpopulation of retinal ganglion cells (RGCs) which are melanopsin-expressing and intrinsically photosensitive (ipRGCs). However, it is still unclear whether photoentrainment of peripheral clocks is mediated exclusively by the ipRGC system or if signaling from RGCs that do not express melanopsin also plays a role. Here we have used genetic “silencing” of ipRGC neurotransmission in mice to investigate whether this photoreceptive system is obligatory for the photoentrainment of peripheral circadian clocks. Genetic silencing of ipRGC neurotransmission in mice was achieved by expression of tetanus toxin light chain in melanopsin-expressing cells (*Opn4*::*TeNT* mouse line). Rhythms of the clock gene *Period 2* in various peripheral tissues were measured by crossbreeding *Opn4*::*TeNT* mice with *PER2* luciferase knock-in mice (*mPER2*^*Luc*^). We found that in *Opn4*::*TeNT* mice the pupillary light reflex, light modulation of activity, and circadian photoentrainment of locomotor activity were severely impaired. Furthermore, *ex vivo* cultures from *Opn4*::*TeNT*, *mPER2*^*Luc*^ mice of the adrenal gland, cornea, lung, liver, pituitary and spleen exhibited robust circadian rhythms of PER2::LUC bioluminescence. However, their peak bioluminescence rhythms were not aligned to the projected LD cycles indicating their lack of photic entrainment *in vivo*. Finally, we found that the circadian rhythm in adrenal corticosterone in *Opn4*::*TeNT* mice, as monitored by *in vivo* subcutaneous microdialysis, was desynchronized from environmental LD cycles. Our findings reveal a non-redundant role of ipRGCs for photic entrainment of peripheral tissues, highlighting the importance of this photoreceptive system for the organismal adaptation to daily environmental LD cycles.

## Introduction

In mammals, the suprachiasmatic nucleus (SCN) in the hypothalamus houses the central circadian pacemaker and orchestrates autonomous circadian oscillations in peripheral tissues to generate coherent rhythms of metabolism and physiology [1–3]. Direct inputs from the retina to the SCN provide the neurochemical signals for its precise alignment to environmental light-dark (LD) cycles [4–6]. This photic information is conveyed by a subpopulation of intrinsically photosensitive retinal ganglion cells (ipRGCs) that express the photopigment melanopsin (*Opn4*) and therefore integrates melanopsin-evoked responses with rod and cone photoreceptor influences [6–9]. In this model, light entrainment of peripheral clocks is reliant on SCN photoentrainment followed by synchronization of peripheral clocks via hormonal/neural mechanisms [10, 11].

Previously, we have demonstrated the essential role of ipRGC glutamatergic neurotransmission in eliciting various NIF visual behaviors such as the pupillary light reflex (PLR) and circadian photoentrainment [12]. However, residual light-evoked NIF behaviors in mice with deficient ipRGC glutamatergic neurotransmission suggested a role of other neurotransmitters with pituitary adenylate cyclase-activating peptide (PACAP) being the most likely candidate [13]. To address whether ipRGC signaling mediates the photic entrainment of peripheral circadian clocks, we examined the circadian rhythmicity of peripheral clocks in mice with “silenced” ipRGC synaptic neurotransmission (*Opn4*::*TeNT*). Blockade of ipRGC synaptic transmission in mice was achieved by selective expression of the tetanus toxin light chain (TeNT) subunit.

Overall, the *Opn4*::*TeNT* mice showed strong suppression of NIF visual behaviors as expected for effective suppression of ipRGC synaptic signaling. PER2::LUC rhythms of peripheral tissues from *Opn4*::*TeNT*, *mPer2*^*Luc*^ mice, including the cornea, were not aligned to the projected environmental LD cycles. Finally, the circadian rhythm in subcutaneous corticosterone, reflecting the functional response of the adrenal clock [14], showed misalignment with environmental LD cycles. Our work in the *Opn4*::*TeNT* mouse thus indicates for the first time that ipRGCs are essential for the temporal alignment of peripheral circadian oscillators to environmental LD cycles.

## Materials and Methods

### Animals

The experimental procedures used in this study were approved by the Institutional Animal Care and Use Committee at the University of Minnesota and are in strict accordance with the recommendations in the Guide for the Care and Use of Laboratory Animals of the National Institutes of Health. Prior to tissue collection, mice were humanely euthanized by isoflurane or carbon dioxide exposure followed by cervical dislocation and all efforts were made to minimize suffering. The animal room was maintained at a 12-h light/dark (LD) cycle (i.e., lights on from 06:00 to 18:00 h). Zeitgeber times (ZTs) of ZT0 and ZT12 were used as the lights-on and lights-off times, respectively. The light intensity at the surfaces of the cages was approximately 500 lux. Mice were fed normal, commercial rodent chow and provided with water *ad libitum*.

### Generation and Breeding Strategy for *Opn4*::*TeNT* mice

We generated *Opn4*::*TeNT* mice by inter-crossing *Opn4*^*Cre/+*^ mice (Cre recombinase inserted in the melanopsin genetic locus)[15] with the *R26*^*floxstop-TeNT*^ mice in which the tetanus light chain subunit (TeNT) is inserted downstream of a *loxP*-flanked STOP sequence into the *R26* locus [16]. *Opn4*::*TeNT* mice (*Opn4*^*Cre/+*^, *R26*^*TeNT/TeNT*^) were compared to control littermates (*Opn4*^*+/+*^, *R26*^*TeNT/TeNT*^) with the exception of multi-electrode array (MEA) and pupillary light reflex measurements where we also included *Opn4*^*+/+*^, *R26*^*TeNT/TeNT*^ and *Opn4*^*Cre/+*^, *R26*^*TeNT/+*^ mice. We used female and male mice in all experiments unless indicated.

### Immunohistochemistry

Immunohistochemistry of brain slices or retinas was performed essentially as described [12]. Briefly, mice were anesthetized with isoflurane and perfused transcardially with 1× phosphate-buffered saline (PBS), pH 7.4, followed by 20 ml of 4% paraformaldehyde in PBS. The brains were extracted and fixed overnight in the same fixative at 4°C. Each brain was sectioned at 100 μm on a Vibratome 1000 (Vibratome, St. Louis, MO). For flat-mount analysis of the retina, the eyes were enucleated and the anterior portion and lens discarded, and the remaining “eyecup” was further fixed in 4% paraformaldehyde in PBS for 1 hr at 4°C. Retinas were isolated then rinsed in PBS (pH 7.4) for 1 hour. Free-floating sections of brain or whole retinas were blocked and permeabilized overnight at 4°C in PBS supplemented with 10% goat serum and 0.5% Triton X-100. The sections were then incubated in primary antibody for two-three days at 4°C, rinsed with PBS, and incubated overnight in secondary antibody at 4°C. After rinsing in PBS for 1 hour, sections were mounted directly to glass slides and covered with the antifade agent Vectashield (Vector Lab, Burlingame, CA). Some retinas were processed as vertical (transretinal) sections using a cryostat (Leica, Buffalo Grove, IL). In this case, the posterior eyecup was immersion fixed in 4% paraformaldehyde in PBS for 15 or 30 min at room temperature. After washing in PBS, the eyecup was cryoprotected in graded sucrose solutions (10, 20, and 30% in PBS) and frozen in embedding medium. Vertical sections (10–20 μm) were cut and collected on superfrost slides. Antibody staining and processing of the retinal sections followed the procedures employed for flat-mount retinas with the exception that the incubation in primary antibodies was abbreviated to an overnight period.

The primary antibodies used in this study were: rabbit anti-melanopsin (1:500)[17], rabbit anti-C-FOS (1:10000)(Calbiochem), mouse anti-glutamine synthetase (1:200) (BD Transduction Laboratories, Franklin Lakes, NJ), goat anti-calretinin (1:200)(Swant, Switzerland), mouse anti-PKC alpha (1:200)(Novus Biologicals, Littleton, CO). We used donkey anti-rabbit Alexa-488 and donkey anti-mouse Alexa-594, donkey anti-goat Alexa-594, as secondary antibodies (Invitrogen, Grand Island, NY, 1:750). Confocal z stacks were acquired using an Olympus FluoView FV1000 confocal microscope with 20X, 40X, or 60X oil-immersion objectives. Editing of images was limited to adjusting the brightness and contrast levels using ImageJ software (NIH, Bethesda, MD). Experiments were replicated with a minimum of 3 mice.

### In Vivo Microdialysis Sampling

Mice under ketamine-xylazine (100–10 mg/kg im) anesthesia were implanted with CMA 20 (10 mm) probes (Harvard Apparatus, Holliston, MA) subcutaneously in the dorsal neck region. Following tethering to a liquid swivel (Instech Labs, Plymouth Meeting, PA), mice were sampled beginning 2 days post-surgery at 60 min intervals for 3 days under a 12:12 h light-dark (LD) cycle. Free corticosterone in dialysate was assayed by ^125^I-corticosterone RIA kit (MP Biomedicals, Santa Ana, CA). A dialysate pool was found to dilute in parallel with the corticosterone standard curve (data not shown); dialysate samples were diluted 1:3 to insure that values fell on the linear part of the standard curve. The *in vivo* microdialysis data were analyzed using the CircWave 1.4 software (Dr. R. Hut, http://www.euclock.org) to test the daily rhythmicity of corticosterone levels and the 24-h rhythm was confirmed if p < 0.05.

### PER2 Luciferase assay

Control and *Opn4*::*TeNT* mice were interbred with *mPER2*^*Luc*^ mice [18]. Mutant mice (*Opn4*^*Cre/+*^, *R26*^*TeNT/TeNT*^, *PER2*^*Luc/+*^) were compared to control mice (*Opn4*^*+/+*^, *R26*^*TeNT/TeNT*^, *PER2*^*Luc/+*^). Mice were sacrificed with CO_2_ asphyxiation/cervical dislocation 2–4 h before lights-off (ZT 8–10). Peripheral tissues (adrenal gland, cornea, lung, liver, spleen and anterior pituitary) were rapidly dissected and placed in ice-cold Hank's solution. Each tissue explant was placed on a membrane (0.4μm, 30 mm in diameter, Millicell cell culture inserts; Millipore, Billerica, MA, USA) in a 35 mm Petri dish, sealed with vacuum grease, and cultured in 1.1 ml DMEM containing 10 mM HEPES, 0.1 mM Luciferin (Promega, Madison, WI), 25 units/ml penicillin, 25 μg/ml streptomycin (Gibco, Grand island, NY), and 2% B27 (Gibco) as described previously [19]. Cultures were incubated at 37°C, and bioluminescence was monitored for 1 min at 7.5-min intervals using a LumiCycle luminometer (Actimetrics, Evanston, IL). Bioluminescence recordings were detrended using a 24 h moving average-subtraction method and smoothed by a 2 h moving average. The first peak in the smoothed data after 24 h *in vitro* was used as a phase marker. Phase angles and variances of circular plots were calculated using Oriana (Kovach Computing Services, Wales, UK).

### Circadian Locomotor Activity

Mice (6–24 weeks of age) were housed within a temperature-controlled facility in individual cages equipped with running wheels that were maintained in ventilated chambers that permitted control of the light-dark cycles. The animals were maintained on a 12-h light (~ 150 lux, white light): 12-h dark (LD 12:12 h) (T24) cycle for at least 3 weeks and then transferred to the ventilated chambers. The intensity of light at 360, 440, 480, 540, 570 and 610 nm was 1.29 · 10^12^, 2.05 · 10^12^, 2.21 · 10^12^, 2.38 · 10^12^, 2.19 · 10^12^ and 2.16 · 10^12^ photons · cm^−2^ · s^−1^, respectively. Irradiance measurements were made with a calibrated radiometer model S370 (UDT Instruments, San Diego, CA) fitted with 10 nm bandpass filters. Manipulation in constant dark (DD) conditions were performed under dim red light. Light negative masking was assessed by recording running wheel activity for mice submitted to a T7 (3.5-h light: 3.5-h dark) cycle [20] over several days. Activity data were recorded continuously by a PC system and displayed and analyzed by using CLOCKLAB software (Actimetrics). The free-running period was calculated (days 1–10 in DD) by using a χ2 periodogram. The amplitude of the circadian component was estimated from a normalized Fourier spectrum by using 10 days in DD. Original data were collected at one-min intervals.

### Intravitreal cholera toxin injections

Mice were anesthetized by intraperitoneal injection of a solution containing ketamine (100 mg/ml) and xylazine (10 mg/ml) at a dose of 0.1 ml /100 g body weight. The vitreous body of the left eye was injected with 2 μl of a 0.1% solution of cholera toxin B protein (CTB) conjugated to Alexa Fluor 488 (ThermoFisher Scientific, Waltham, MA) in sterile PBS. Following the injection, the needle was held in place for 30 sec to prevent leakage. The same procedure was repeated for the right eye with injection of CTB conjugated to Alexa Fluor 594. Mice were perfused with 4%oParaformaldehyde in PBS intracardially 2 days after the injection. Brain were processed and imaged as described above for immunohistochemistry.

### Electrophysiology

After removal from the eye, a patch of retina about 4–10 mm^2^ was mounted on a Multi-electrode array (Multichannel Systems, Reutlingen, Germany), ganglion cell side down, and perfused with oxygenated Ames’ medium at 35°C supplemented with 20 μM CNQX ((6-cyano-7-nitroquinoxaline-2,3-dione)) and 50 μM D-APV (D(−)-2-Amino-5-phosphonopentanoic acid) to block glutamatergic transmission. The activity of ganglion cells was recorded via 256 electrodes 30 μm in diameter spaced every 100 μm apart and arranged in a 16×16 square grid (Multi Channel Systems MCS GmbH). Full-field visual stimuli were presented during recordings using a high brightness LED (LuxeonStar 5, luxeonstar.com) with a peak wavelength of 480 nm. The light stimulation protocol consisted of 1min stimuli of increasing irradiance (5.10^10^ photons/cm^2^/s to 5.10^13^ photons/cm^2^/s). The current through the LED was controlled using custom electronics and software written in Matlab (Mathworks, Natick, MA) and aligned with the physiological recording with a resolution of ±100 μs. Signal is acquired from all 256 channels @ 10 kHz. Negative thresholds for spike detection were set at 5 times the standard deviation of the noise on each channel. Spike cutouts, consisting of 1 msec preceding and 2 msec after a suprathreshold event, along with a time stamp of the trigger were written to hard disk. For each electrode, these spike cutouts were sorted into trains of a single cell after recording using Offline Sorter (Plexon, Denton, TX). Data analysis and display were performed using Neuroexplorer (Plexon) and custom software written in Matlab.

### Pupillary Light Reflex (PLR) Measurements

Mice were implanted with an acrylic headpost. After at least 1 week of recovery from the headposting surgery, they were tested for PLR. On the day of the testing, 15 min before the light stimulation, dark-adapted mice received a drop of tropicamide in the left eye. Before the recordings, mice were briefly anesthetized with isofluorane and restrained in a custom-made animal holder. The animal holder was placed inside a light tight box with the left eye apposed against an opening of an integrating sphere. Light from a 300 Watt Xenon Arc lamp light source (Sutter Instrument, Novato, CA, USA) was filtered, collimated and delivered to the integrating sphere through a liquid light guide. An inline 480 nm filter, a filter wheel with a neutral density filter and a Lambda 10–3 optical filter changer with SmartShutter^TM^ were used to control the spectral quality, intensity and duration of light. Light intensity was measured with apower meter (Melles Griot, Rochester, NY). The mouse’s right eye was illuminated by an IR LED and recorded with a high precision LINX video camera (Imperx Inc., Boca Raton, FL) equipped with an IR filter at a sample rate of 7Hz. We recorded sequences consisting of 1 min of darkness, 1 min of monochromatic 480 nm light (1.10^10^ photons/cm^2^.s and 1.10^14^ photons/cm^2^.s) and finally 3 min of darkness. Digital movies of pupil constriction were analyzed with a custom Matlab (Mathworks®) program. We extracted the pupil diameter. The mean diameter measured during the first period of darkness (1 min) of each sequence served as the baseline for normalization of the recordings.

### Statistical Analyses

Statistical analyses were performed using OriginPro 8.1 (Origin Lab, Northampton, MA) or GraphPad InStat (GraphPad, LaJolla, CA). Statistical comparison of means was performed using a Student's t test or one-way analysis of variance (ANOVA) followed by Tukey–Kramer multiple comparison tests. In some cases, comparison of means were performed by Kruskal-Wallis non-parametric ANOVA, followed by Dunn’s multiple comparison test. Data are presented as mean ± SE.

## Results

### Synaptic “silencing” of ipRGCs impairs PLR and decreases light-evoked c-FOS expression in the SCN.

To suppress ipRGC neurochemical signaling, we used targeted expression of tetanus toxin light chain fragment (TeNT) on ipRGCs. TeNT inhibits chemical neurotransmission by cleaving the synaptic vesicle membrane protein VAMP2/Synaptobrevin-2 with consequent decrement or suppression of synaptic signaling of neurons that express it [21]. Selective expression of TeNT in ipRGCs was achieved by breeding *Opn4*^*Cre/+*^ mice in which *Cre* recombinase is inserted in the melanopsin (*Opn4*) genetic locus [15] with *R26*^*floxstop-TeNT*^ mice in which expression of the TeNT is dependent on Cre recombinase activity [16]. Mutant mice (*Opn4*^*Cre/+*^, *R26*^*TeNT/TeNT*^) r, showed no apparent abnormalities during development although an extensive assessment of their behavior or anatomy has not been carried out.Retinas of adult *Opn4*^*Cre/+*^, *R26*^*TeNT/TeNT*^ mice had an apparent normal morphology with comparable thickness of the outer nuclear and inner nuclear layers (S1 Fig). Immunostaining for various immunocytochemical markers (calretinin, Protein Kinase C-α, glutamine synthetase) revealed similar expression in the mutant mice (S1 Fig). Melanopsin staining in whole mount retinas or retinal sections showed the presence of ipRGCs including the outer stratifying M1 cells, inner stratifying M2 and displaced M1 cells. Comparable numbers of melanopsin-positive retinal ganglion cells were found in the control (*Opn4*^*+/+*^, *R26*^*TeNT/TeNT*^) and mutant (*Opn4*^*Cre/+*^, *R26*^*TeNT/TeNT*^) retinas (S2 Fig). Although TeNT is fused to GFP, we failed to detect the presence of GFP in the mutant retinas possibly due to the low levels of expression of TeNT. Injection of fluorescently conjugated cholera toxin into the eye, which labels all ganglion cell fibers from the retina, showed that fibers that innervated NIF visual targets such as the SCN (S3 Fig), intergeniculate leaflet (IGL) and ventral lateral geniculate nucleus (vLGN) were preserved in the *Opn4*^*Cre/+*^, *R26*^*TeNT/TeNT*^ mice (S3 Fig).

**Fig 1:**
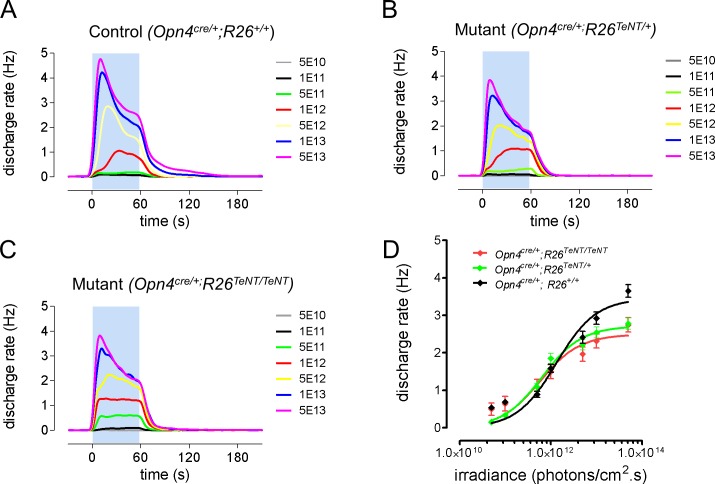
TeNT expression doesn’t alter ipRGCs responses to light. (A-C) Average ipRGCs responses to 1min light stimulations (480nm) of increasing irradiance (5.10^10^ photons/cm^2^/s to 5.10^13^ photons/cm^2^/s) recorded from retinas of *Opn4*^*cre/+*^, *R26*^*+/+*^ (A, n = 123), *Opn4*^*cre/+*^, *R26*^*+/TeNT*^ (B, n = 87), *Opn4*^*cre/+*^, *R26*^*TeNT/TeNT*^ (C, n = 158) mice and the corresponding dose response curves (D).

We examined the light-evoked responses of ipRGCs in isolated retinas of mutant and control mice using multi electrode array (MEA) recordings (Fig 1). We recorded from early postnatal mice given that ipRGCs are the only light responsive cells at this developmental stage [22, 23]. As expected, we observed light responses with increasing firing rates with higher levels of light stimulation. During the 60 second period of light simulation the firing rate at higher light intensities reached a maximum followed by substantial progressive decline. Upon termination of the light stimulus, ipRGCs continued to display substantial spike activity that persisted for several seconds. The relatively sluggish initiation and termination of spike responses are consistent with previous reports of melanopsin-evoked light responses [23]. Overall there were no significant differences across genotypes indicating that expression of TeNT in ipRGCs did not impact their overall responsiveness to light.

**Fig 2:**
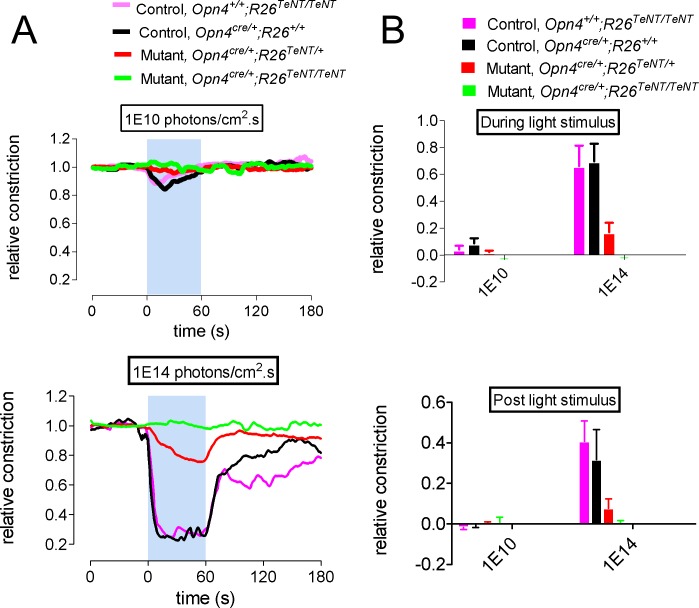
Synaptic “silencing” of ipRGCs and loss of pupillary light reflex (PLR) in Opn4::TeNT mice. (A) Opn4Cre/+ mice were intercrossed with R26floxstop-TeNT mice that have the TeNT gene integrated into the Rosa26 locus downstream of a floxed transcriptional stop sequence. Expression of Cre recombinase in ipRGCs excises the stop sequence to activate TeNT expression irreversibly. (B) Representative images of pupillary constriction 20 s post-irradiation in control and Opn4::TeNT mice. White broken lines encircle pupillary diameters. (C) Relative pupil area upon light stimulation of control and Opn4::TeNT mice with low and high light intensities. Values denote means ± SEM (n  =  3–5). *p<0.001.

To examine whether expression of TeNT in ipRGCs suppressed NIF behaviors, we measured consensual PLR at two light intensities on awake control and mutant mice. Following a four-hour dark period adaption, PLR was evoked by a 60 second 480 nm light stimulus and pupil responses were recorded in the contralateral eye using a low-light video camera. In control littermate mice (*Opn4*^*+/+*^, *R26*^*TeNT/TeNT*^ and *Opn4*^*+/+*^, *R26*^*+/+*^) the pupils constricted both at low (1X10^10^ photons/cm^2^.s) and high (1X10^14^ photons/cm^2^.s) light intensities (Fig 2A and 2B). In contrast, *Opn4*^*Cre/+*^, *R26*^*TeNT/TeNT*^ mice had a significant deficit of PLRs even at high light intensities as reflected by the relatively small constriction of pupils (normalized pupil constriction: ctrl *Opn4*^*+/+*^, *R26*^*TeNT/TeNT*^: 0.66 ± 0.15; mut: -0.01 ± 0.01; p<0.001, unpaired t-test). Interestingly, an intermediate level of pupil constriction (0.17 ± 0.07) was observed in the *Opn4*^*Ccre/+*^, *R26*^*TeNT/+*^ mice indicating a gene dosage effect on the TeNT-mediated synaptic silencing of ipRGCs Lack of light-evoked responses in the *Opn4*^*Cre/+*^, *R26*^*TeNT/TeNT*^ was not due to malformation of the pupillary constriction apparatus as topical application of 100 mM carbachol in the mutant mouse eye (n  =  3) was able to elicit full pupillary constriction. Thus we conclude that PLRs are deficient in the *Opn4*^*Cre/+*^, *R26*^*TeNT/TeNT*^ mice in accordance with previous observations on the role of ipRGCs in this particular NIF behavior [24, 25]. Based on these results we used the *Opn4*^*+/+*^, *R26*^*TeNT/TeNT*^ as control mice and the *Opn4*^*Cre/+*^, *R26*^*TeNT/TeNT*^ as mutant mice and referred as *Opn4*::*TeNT* hereafter.

**Fig 3:**
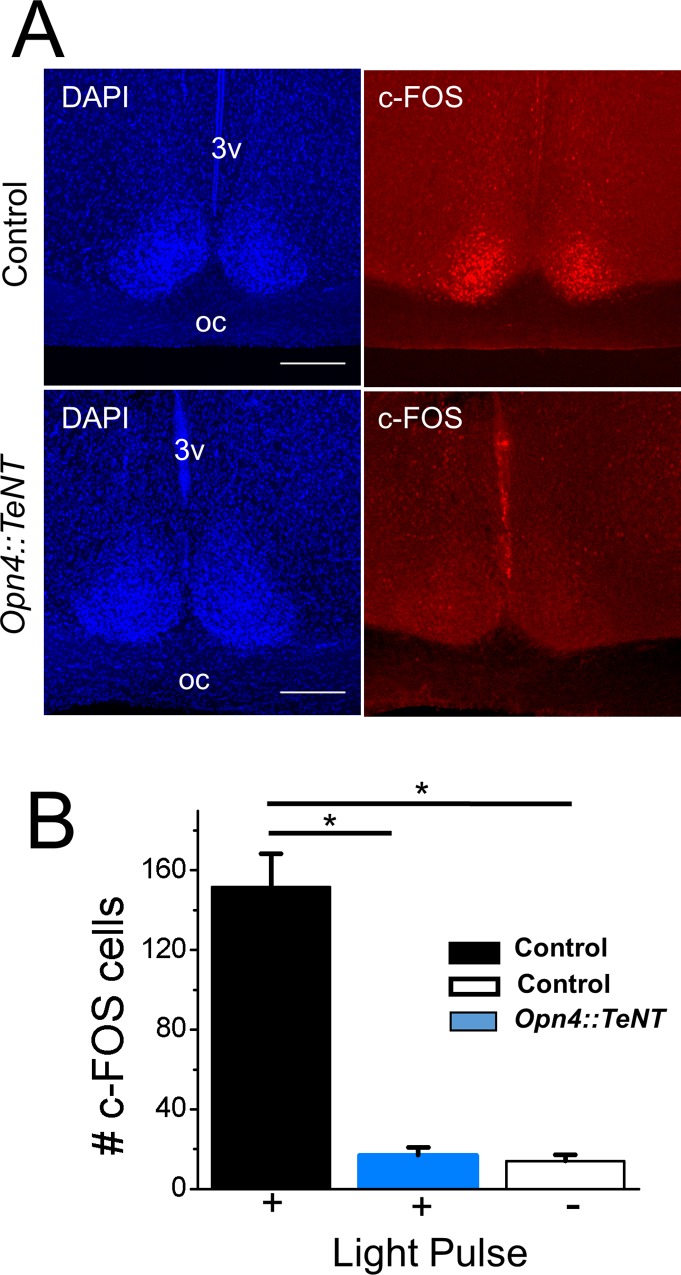
Lack of c-FOS expression in the *Opn4*::*TeNT* suprachiasmatic nucleus following light pulse at CT 16. (A) Immunocytochemical staining for c-FOS in the SCN of control and *Opn4*::*TeNT* mice exposed to a light pulse. Notice the diminished number of c-FOS positive cells in the mutant mouse. Scale bar = 100 um. (B) Quantification of c-FOS expression in the suprachiasmatic nucleus in control mice exposed to a light pulse (+) or no light pulse (-) and in mutant mice exposed to a light pulse. Expression is reflected by the number of c-FOS positive cells per suprachiasmatic nucleus per slice. n = 3–8. *P<0.004 by Kruskal-Wallis non-parametric ANOVA, followed by Dunn’s multiple comparison test.

The immediate early gene c‐Fos has been used as a marker of light‐induced neuronal activity in SCN neurons with marked increase in its expression with light pulses delivered at subjective night [26, 27]. Because the *Opn4*::*TeNT* mice did not show light entrainment in locomotor activity (see following section), we used the onset of locomotor activity at circadian time (CT)12 as a phase reference point for both control and mutant mice. Mice were kept in constant dark (DD) for two days and then a 30 min light pulse was administered at CT16; mice were kept in DD for 60 min until perfusion. Using immunohistochemistry, we confirmed that c-FOS as measured by the number of immunopositive cells is induced in the SCN of control mice (n = 151.7 ± 16.6)(Fig 3). By comparison, the *Opn4*::*TeNT* mice showed significantly lower levels of induction of c-FOS within the SCN (n = 16.8 ± 4.0) (p<0.001, One-way ANOVA, Bonferroni's post hoc test for pairwise comparison); which was not significantly different from sham control mice (n = 14.0 ± 3.2)(p>0.05, One-way ANOVA, Bonferroni's post hoc test for pairwise comparison). These findings indicate the lack of ipRGC neurochemical signaling to the SCN in the *Opn4*::*TeNT* mice.

### Synaptic “silencing” of ipRGCs leads to lack of photoentrainment in locomotor activity and absence of light negative masking

**Fig 4:**
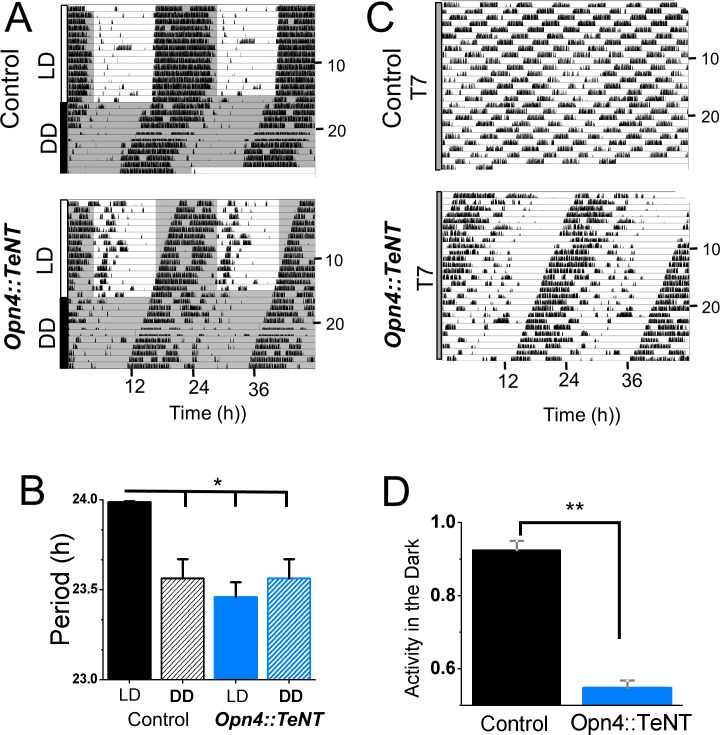
Impaired photoentrainment and light negative masking in *Opn4*::*TeNT* mice. (A) Representative activity records from individual animals initially maintained under a 12:12 h LD cycle regimen for >2 weeks and then transferred to DD conditions for 2 weeks. A control animal demonstrates light entrainment in LD as indicated by enhanced activity in the dark period while the *Opn4*::*TeNT* mouse shows activity that follows a “free running” pattern. Shaded gray regions indicate periods of darkness. (B) Summary of measured periods (Tau) in control and *Opn4*::*TeNT* mice under LD and DD conditions. n = 12–13. (C) Activity records of control and *Opn4*::*TeNT* mice kept in ultradian light cycles of 3.5:3.5 light dark periods (T7). (D) Control mice are active mostly during dark periods under T7 conditions while the *Opn4*::*TeNT* mice are active across the light and dark periods. n = 4–5. *p<0.01. **; **p<0.001.

Behavioral analysis of circadian locomotor activity was carried out to ascertain whether *Opn4*:*TeNT* mice align their activity to LD cycles. In these experiments, mice were individually housed in cages containing a running wheel, and their daily locomotor activity was recorded. The animals were initially exposed to regular LD cycles (12:12 h). In these conditions, the control mice showed normally entrained daily patterns of activity, initiating their nightly bouts of activity at the beginning of the dark period and exhibited nocturnal rhythms in activity as reflected by the period length (tau) of 23.98 ± 0.01 (n = 12)(Fig 4A and 4B). All *Opn4*::*TeNT* mice, however, recorded in LD 12:12 conditions failed to entrain their circadian rhythms to external light cues and showed a “free running” rhythm with a tau of 23.46 ± 0.08 (n = 12). There was no difference in free-running periods of *Opn4*::*TeNT* mice kept on LD 12:12 or DD conditions (tau = 23.56 ± 0.11, n = 12)) (p>0.05, One-way ANOVA, Bonferroni's post hoc test for pairwise comparison) suggesting that the SCN circadian clock in these animals oscillates independently of external light conditions.

Suppression of locomotor activity in nocturnal rodents by light (light negative masking) is another NIF behavior associated with light signaling by ipRGCs [25]. This behavior was assessed by exposing the mice to an ultradian light regimen of 3.5:3.5 light/dark cycles (T7) for two to three weeks (Fig 4C). This light regimen measures the effects of light independent of circadian rhythms as the mice fail to entrain to light cycles that move across the circadian cycle [28]. Activity in the dark was normalized to the total activity to yield the relative activity in the dark. The control group exhibited a relative activity in the dark of 0.92 ± 0.02 indicating a strong bias for activity to the dark periods. The mutant group, however, showed a relative activity in the dark of 0.55 ± 0.02 consistent with near equal activity during the light and dark periods (Fig 4C and 4D).

### PER2::LUC rhythms in peripheral tissues do not entrain to light in the *Opn4*:*TeNT* mice.

**Fig 5:**
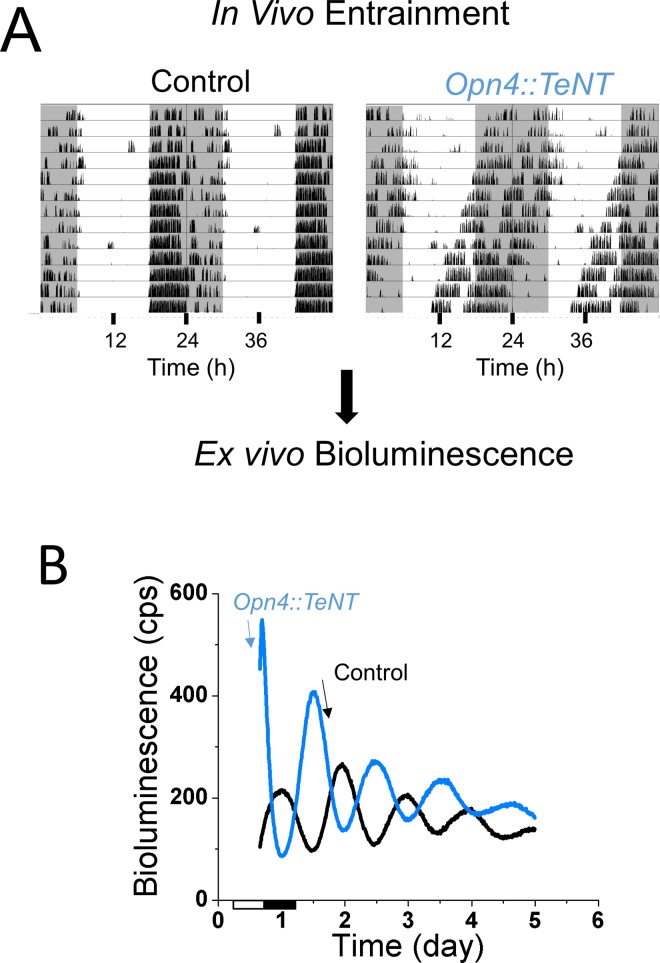
PER2::LUC rhythms are maintained in peripheral tissues of *Opn4*::*TeNT* mice. (A) Examples of locomotor activity in a control and a *Opn4*::*TeNT* mouse on a 12:12h LD cycle. Locomotor activity was recorded using running wheels and animals were sacrificed when activity was phase shifted by 8–12 hours between control and *Opn4*::*TeNT* mice. Tissues were then collected for ex-vivo bioluminescence. (B) Examples of cornea PER2::LUC bioluminescence rhythms in *Opn4*::*TeNT* (blue) and control (black) mice. Notice the antiphasic peak of bioluminescence in the mutant cornea compared to the cornea from a control mouse.

**Fig 6:**
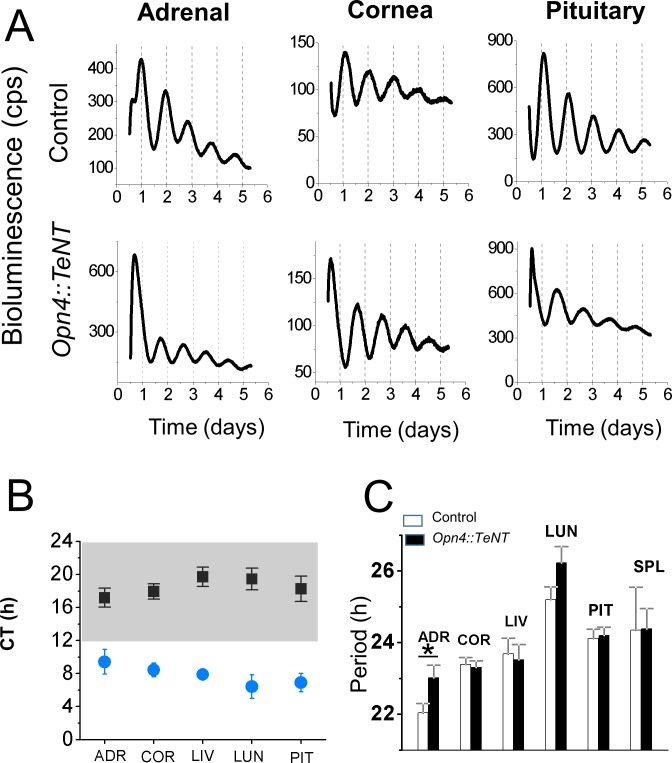
PER2::LUC rhythms are out of phase to LD cycles. (A) Representative records of bioluminescence from various peripheral tissues in control and *Opn4*::*TeNT* mice with access to running wheels. (B) Phases for central and various peripheral circadian oscillators of control and *Opn4*::*TeNT* mice. The peak of the circadian oscillation was determined during the interval between 12 and 36 h in culture. The average times (± SEM) of peaks were plotted against the projected CT time. Notice that in the control mice the peak of PER2::LUC expression occurs in peripheral tissues during the subjective night (CT12-24). On the other hand, in the *Opn4*::*TeNT* mice the peak of PER2 expression is aligned to subjective day (CT0-12). (C) Period values of PER2::LUC rhythms were comparable in control and *Opn4*::*TeNT* mice with the exception of period lengthening in the adrenal. ADR, adrenal; COR, cornea; LIV, liver; LUN, lung; PIT, anterior pituitary; SPL, spleen. *P<0.05. ADR, adrenal (n = 12–17); COR, cornea (n = 14–22); LIV, liver (n = 12–14); LUN, lung (n = 13–14); PIT, anterior pituitary (n = 15–20).

Robust self-sustaining PER2::LUC circadian rhythms have been measured *in vitro* for the SCN and peripheral tissues using the *mPer2*^*Luc*^ knockin mice [18]. In these mice, PER2::LUC rhythms measured *in vitro* likely reflect the *in vivo* circadian rhythms of PER2 protein [29]. Thus to examine the effect of ipRGC silencing on the photic entrainment of peripheral circadian oscillators, we crossed the *Opn4*::*TeNT* with *mPER2*^*Luc*^ mice. We kept the mutant and control mice on regular LD cycles (12:12 h) with free access to running wheels for several days and sacrificed the mice when their circadian locomotor activity became approximately antiphasic (8–12 h phase difference) (Fig 5A). Under these conditions, explant cultures of peripheral tissues from mutant and control mice showed robust rhythms of PER2::LUC that were approximately antiphasic (Fig 5B and Fig 6A). Similar to what has been previously reported [18], peripheral tissues of control mice displayed prominent circadian oscillations in PER2::LUC bioluminescence with a peak in the dark phase of the cycle between CT 17–20 (Fig 6B). However, explant cultures from the *Opn4*::*TeNT* mice showed peak PER2::LUC expression in the light phase of the cycle between CT 7–10 suggesting the lack of photoentrainment of peripheral tissues *in vivo* (Fig 6B). The average period for all tissues were comparable in control and mutant mice (p>0.05, unpaired two-tailed Student’s t-test), with the exception of adrenals (p = 0.03, unpaired two-tailed Student’s t-test) (Fig 5C).

**Fig 7:**
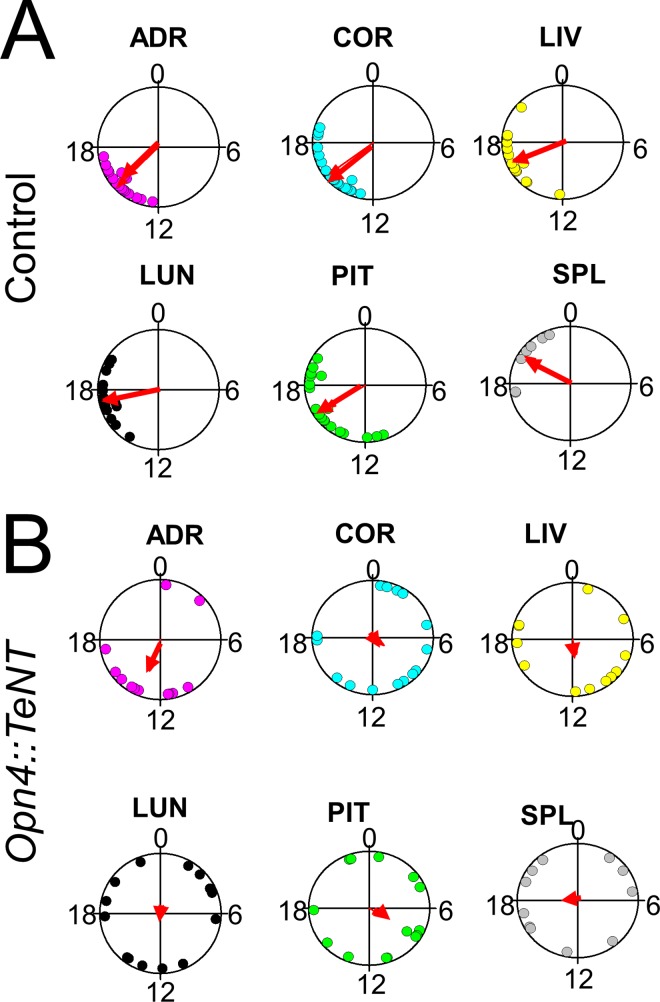
PER2::LUC rhythms of peripheral tissues in *Opn4*::*TeNT* mice are not entrained by light. Circular plots of peak bioluminescence rhythms in peripheral tissues presented in control (A) and mutant (B) mouse tissues. Mice were housed in the absence of running wheels. Notice the phases of peak bioluminescence rhythms are significantly dispersed among tissues in the *Opn4*::*TeNT* mice indicating their lack of entrainment to environmental light-dark cycles. ADR, adrenal (n = 12–17); COR, cornea (n = 15–20); LIV, liver (n = 12–14); LUN, lung (n = 14–16); PIT, anterior pituitary (n = 14–19; SPL, spleen (n = 7–11).

In order to further assess the effects of light on the circadian rhythmicity of peripheral tissues, we also housed the animals in LD cycles (12:12 h) in the absence of running wheels. We reasoned that in control mice the peripheral tissues should maintain their circadian PER2::LUC rhythms in phase to light-dark cycles and in phase to each other. The *Opn4*::*TeNT* mice on the other hand were expected to show circadian oscillations independent of LD cycles with no phase relationships to each other. Analysis of peak PER2::LUC expression using phase coherence maps of the peak time on the second day show clustered expression of PER2::LUC rhythms in all tissues examined in the control mice (Fig 7) (Rayleigh uniformity test, P < 0.001). However in the tissues derived from *Opn4*::*TeNT* mice, there was considerable dispersion of the peak times for each tissue examined with a low degree of phase coherence (Rayleigh uniformity test, P > 0.05) with exception of adrenals (Rayleigh uniformity test, P = 0.02). These results suggest that light failed to serve as an entrainer for the peripheral tissues examined in the absence of ipRGC neurochemical signaling.

### Circadian glucocorticoid release is not synchronized to LD cycles in the *Opn4*::*TeNT* mice

**Fig 8:**
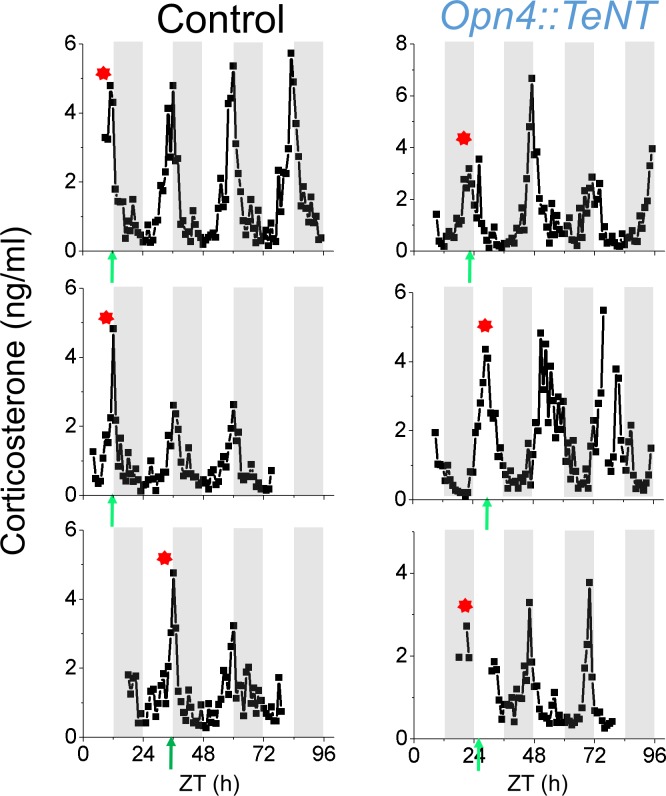
Circadian corticosterone secretion in *Opn4*::*TeNT* Mice is not entrained to LD Cycles. Free corticosterone measured in extracellular fluid by *in vivo* subcutaneous microdialysis in 3 control mice and 3 *Opn4*::*TeNT* mice maintained under 12:12 h LD cycles. Shaded gray regions indicate periods of darkness. Arrows indicated projected CT12 as assessed by running wheel activity that preceded the microdialysis sampling procedure. Subcutaneous free corticosterone in control mice shows circadian rhythms that peak at the onset of the dark period and the projected CT12. In the *Opn4*::*TeNT* mice the corticosterone rhythms are maintained, but are not aligned to the LD cycles. Notice however that in the mutant mice the peak of the corticosterone rhythm is aligned to the projected CT12. Corticosterone CircWave peak phases are indicated with (*).

In nocturnal rodents, baseline plasma corticosterone concentrations vary predictably peaking immediately before the onset of activity [30]. Thus we asked the question whether the circadian pattern of corticosterone in mice is entrained by light in an ipRGC-dependent manner. Mice were housed in a 12:12 h LD cycle in cages containing running wheels for three weeks before the implantation of the microdialysis probe. The LD schedule was then maintained during the microdialysis sampling in cages without running wheels. Subcutaneous corticosterone, which is highly synchronized to plasma corticosterone rhythms in rats [31], was measured at 1-hour intervals for two-three days using *in vivo* microdialysis. The periodicity and phase of corticosterone rhythms in dialysate was assessed using CircWave software which aims to produce a Fourier-curve that describes the data by adding as many harmonics to the wave as the data allow(Fig 8). In both experimental groups, the subcutaneous corticosterone showed a circadian rhythm (CircWave, p<0.0001)) with peak times aligned to the expected onset of running wheel activity. However, whereas the corticosterone peak aligned with the beginning of the dark phase in the control group, the mutant animals showed a peak corticosterone that was not synchronized to LD cycles (Fig 8). Taken together, our data show that the lack of ipRGC signaling prevents synchronization of corticosterone rhythms to environmental LD cycles.

## Discussion

Circadian rhythms in the function of virtually every cell type allows anticipatory adaptive responses to best cope to daily changes in LD cycles, food intake and locomotor activity [2]. Traditionally, the circadian system has been modeled as a hierarchical system with a central circadian pacemaker in the SCN and “subordinate” peripheral oscillators in tissues throughout the body [2, 32, 33]. Since the period of biological oscillators or clocks is circadian (approximately but not exactly 24 h), the clocks are entrained daily by encoding signals such as environmental LD cycles to maintain synchrony with the environment. [13]. In mammals, light entrainment signals require ipRGCs to convey photic signals to the SCN [6, 34], since mice which lack ipRGCs have normal visual acuity but are not entrained by light [25]. In the simplest hierarchical model of circadian photoentrainment, the SCN is entrained to LD cycles via ipRGC input from the retina that in turn sends efferent signals to synchronize peripheral clocks for the temporal alignment of physiological rhythms. This notion is strongly supported by findings that selective ablation of the SCN leads to a complete loss of circadian rhythmicity, whereas transplantation of an intact SCN into arrhythmic mutant animals restores circadian rhythmicity [35, 36]. However, the recent development and description of forebrain- or SCN-specific knockout mice for the essential clock gene *Bmal1* [37–39] raises important questions. In these mice, peripheral oscillators are able to sustain circadian rhythmicity that are phase-locked with external LD cycles, even in the absence of a functional central SCN oscillator [37–39]. These results suggest that a functional SCN clock is not required for entrainment of peripheral clocks and that light can directly entrain peripheral tissues via brain clocks other than the SCN [11, 39].

In this study we evaluated the role of ipRGCs in light entrainment of peripheral clocks using a genetic approach. We used a mouse model in which synaptic communication between ipRGCs and target neurons in the brain is reduced or eliminated. Such an approach has been used with success in other neuronal types [16, 40, 41] and was chosen because our attempts to remove glutamatergic transmission in ipRGCs proved to be only partially successful in blocking NIF responses [12]. The *Opn4*::*TeNT* mouse, on the other hand, displayed near complete suppression of NIF responses such as the PLR and light-suppression of locomotor activity. The differences between the ipRGC-specific knockout for glutamatergic neurotransmission [12] and the *Opn4*::*TeNT* mouse is likely explained by the residual release of other neurotransmitters from ipRGC terminals such as the pituitary adenlylate cyclase-activating peptide (PACAP) which is found in melanopsin-containing cells [42, 43]. Presumably, expression of tetanus toxin fragment in ipRGCs suppressed the synaptic release of both glutamate and PACAP. In general terms the *Opn4*::*TeNT* mouse phenocopies the genetically ablated ipRGC mouse lines via diphtheria toxin expression [24, 25] in regard to circadian photoentrainment and PLR. The TeNT approach has the advantage that the genetically targeted ipRGCs are still present, they receive synaptic contacts and therefore is less likely to induce large perturbations in the retina. Residual electrical signaling of ipRGCs in the *Opn4*::*TeNT* mouse to other neurons cannot be discarded given recent reports of ipRGC to amacrine cell gap junctional coupling [44]. However, we don’t find evidence that this form of ipRGC signaling play a major role in PLRs, light negative masking, and circadian photoentrainment given the almost complete ablation of these NIF functions in the *Opn4*::*TeNT* mouse. Finally, because Cre recombinase in the *Opn4*::*TeNT* mouse line is expressed according to historic patterns of melanopsin expression, it remains a possibility of “silencing” RGCs that only transiently express melanopsin during development. We don’t think, however, this is very likely as it has been shown that more than 90% of Cre positive cells harbor intrinsic melanopsin photosensitivity in the Opn4-Cre mouse line employed in our study [15].

Overall, the lack of neurochemical signaling from ipRGCs did not markedly influence the circadian locomotor activity. The average tau for circadian locomotor activity for the *Opn4*::*TeNT* mice held in DD or LD was almost identical to the average tau of control mice in DD conditions. Thus the periodicity of the circadian oscillator in the SCN that controls circadian locomotor activity was not affected by the synaptic “silencing” of melanopsin-containing photoreceptors. Light masking of locomotor activity was, however, absent in the mutant mice as disclosed in animals under short ultradian light cycles in line with previous findings that suppression of activity by light is primarily dependent upon ipRGC signaling [25]. It has been suggested that in addition to the classical retinohypothalamic pathway that directly entrains the SCN clock, a secondary neural pathway bypassing the SCN clock could relay photic signals for synchronization of peripheral clocks [11]. Our results implicate ipRGCs as the sole source of photic signals to peripheral clock synchronization either via the classical retinohypothalamic pathway or the putative SCN clock bypassing pathway

Importantly, using in vitro PER2Luc activity as a readout of circadian rhythmicity *in vivo*, we found that in peripheral tissues from *Opn4*::*TeNT* mice, the peripheral clocks are not synchronized to ambient LD cycles. Instead, they followed their predicted endogenous circadian rhythms as measured by running wheel activity. The peripheral tissues (adrenal gland, cornea, lung, liver, anterior pituitary, and spleen) maintained robust circadian oscillations of PER2::LUC in the *Opn4*::*TeNT* mice with average tau comparable to control animals. Contrary to a previous report showing resetting of the corneal circadian clock following dissection [45], the corneal rhythms seemed to follow the animals’ endogenous circadian time in our experiments. PER2Luc activity of corneal tissues from control and *Opn4*::*TeNT* mice sacrificed at ZT8-10 were approximately antiphasic, as predicted from their circadian locomotor activity (Fig 5B and Fig 6). The lack of light entrainment of corneal tissue in the *Opn4*::*TeNT* mice is not consistent with previous studies showing that corneal tissue is entrained directly by light *in vitro* without contribution of rods, cones or ipRGCs perhaps via neuropsin signaling [46]. In the retina and cornea, light might act directly on these ocular tissues to regulate circadian phase [46, 47]. However, it is possible that humoral entrainment signals to the cornea *in vivo* override neuropsin mediated influences. A candidate for this humoral signal are the glucocorticoids secreted by adrenal glands which are rhythmically released and contribute to synchronization of the cell-autonomous clocks in the body [48–50]. Another potential entrainer of corneas *in vivo* is melatonin given that melatonin entrains circadian rhythms of corneas *in vitro* [51], however this was not investigated here as mice employed in our study are likely to be deficient in melatonin production[52]. Finally, it is also plausible that under another set of light intensities or spectral characteristics the contribution of neuropsin to light entrainment of cornea may become more significant. We have not systematically varied light intensities and spectral properties in our experiments so we cannot discard such possibility.

To investigate the effect of silencing ipRGC signaling on adrenal function, we monitored corticosterone rhythms using subcutaneous microdialysis sampling. Due to the enhanced temporal resolution of this approach, we were able to assess phase relationships between peak corticosterone and the light-dark cycle to directly test whether photic entrainment of the corticosterone rhythm requires ipRGC signaling. In control mice, the peak of the corticosterone rhythm occurred near the onset of the dark period, supporting previous work in rats [33]. In *Opn4*::*TeNT* mice, however, the peak of the corticosterone rhythm does not occur at the onset of the dark phase, but remains aligned with the locomotor activity rhythm. These results provide direct evidence that photic entrainment of the corticosterone rhythm is dependent on ipRGC signaling. Previous work in rodents has used light exposure during the dark period to indirectly infer the role of ipRGCs in the control of corticosterone release. By filtering out blue light, which optimally drives melanopsin phototransduction, light-induced increases in corticosterone and adrenal clock gene expression in rats were prevented (Rahman et al., 2008). Conversely, exposure to blue light in mice increases plasma corticosterone and gene expression in adrenals more efficiently than longer wavelengths of light [53] and the effects of blue light were attenuated in melanopsin knockout mice [53]. These published findings are consistent with the data presented here, in which blocking ipRGC neurotransmission prevented photic entrainment of corticosterone rhythms in mice.

Light-induced increases in cortisol also have implicated ipRGC signaling in control of adrenal activity in humans. Both light-induced suppression of plasma melatonin and increases in plasma cortisol are prevented by filtering out short wavelength (blue) light [54]. By exposing individuals to narrowband blue or red light, Figueiro and Rea examined the effects of light on salivary melatonin and cortisol in humans [55]. Blue light was highly effective in reducing melatonin secretion. In contrast, both red and blue light increased nocturnal release of cortisol [55], suggesting that a melanopsin-independent pathway may contribute to control of adrenal corticosteroid secretion in humans. It is possible that different ipRGC subtypes play distinctive roles in different species with the melanopsin-rich M1 type [56, 57] more relevant to adrenal photoentrainment in mice and the non-M1 subtypes which are more influenced by outer retina photoreceptors [57] exerting a more prominent role in humans, although there is no experimental evidence yet that in primates, M1-like and M2-like ipRGCs respond to light differently.

In conclusion, we report here that ipRGC signaling serves as a critical element for light entrainment of various peripheral clocks. Blockade of neurotransmitter signaling from ipRGCs in mice leads to a free running behavior of the peripheral tissues in regard to clock gene expression and glucocorticoid rhythms. It expands the large number of adaptive responses to light mediated by ipRGCs and underscores the essential role of this photoreceptive system as a regulator of circadian rhythmicity.

## Supporting Information

S1 Fig Immunofluorescence labeling for glutamine synthetase (GS), PKC-α, and calretinin in control and *Opn4*::*TeNT* retinas. Retinas were also counterstained with DAPI. Notice the comparable thickness of cell layers and similar pattern of expression for GS, PKC-α, and calretinin. Similar staining was observed in other two mice in each group. GCL, ganglion cell layer, INL, inner nuclear layer, ONL, outer nuclear layer. Scale bar = 50 μm.
(TIF)

S2 Fig Expression of melanopsin in control and *Opn4*::*TeNT* retinas. (A) Immunostaining for melanopsin in whole-mount retinas of control and *Opn4*::*TeNT* retinas. Confocal images at retinal ganglion cell layer reveals the presence of melanopsin-expressing cells in control and mutant mice retinas. (B) Similar densities of melanopsin expressing cells in the retinal ganglion cell layer of control and *Opn4*::*TeNT* retinas (n = 6). Scale bar = 100 μm.
(TIF)

S3 Fig Projections of RGCs to the SCN and LGN in control and *Opn4*::*TeNT* mice. The fidelity of RGC central projections in control and *Opn4*::*TeNT* mice adult mice was assessed by anterograde fiber tracing. Mice were killed and perfused, and brain sections analyzed by fluorescence microscopy after injection of Alexa 594–conjugated cholera toxin β into the right eye and Alexa 488–conjugated cholera toxin β into the left eye. Right eye (red) and left eye (green) RGC projections to the SCN (A) and LGN (B). Oc, optic chiasm; dLGN, dorsal LGN; IGL, intergeniculate leafleat; vLGN, ventral LGN. Scale bar = 100 μm for (A) and 200 μm for (B).
(TIF)
