# Supplementary figures and images for: Intrinsically Photosensitive Retinal Ganglion Cells (ipRGCs) Are Necessary for Light Entrainment of Peripheral Clocks
Source: PLoS One. 2016 Dec 16;11(12):e0168651. doi: 10.1371/journal.pone.0168651 (PMC5161485; doi:10.1371/journal.pone.0168651)

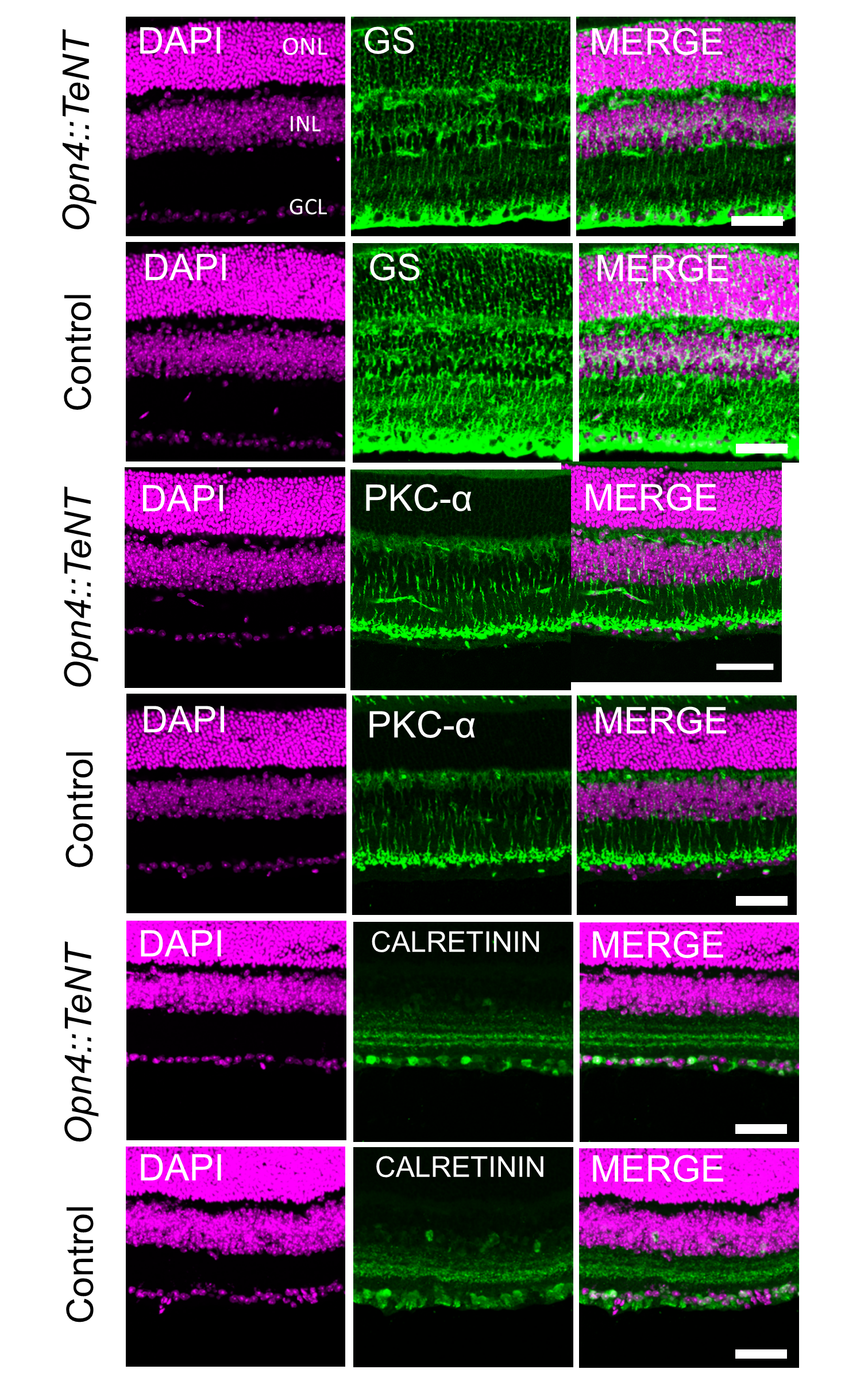

Supplement: S1 Fig — Retinas were also counterstained with DAPI. Notice the comparable thickness of cell layers and similar pattern of expression for GS, PKC-α, and calretinin. Similar staining was observed in other two mice in each group. GCL, ganglion cell layer, INL, inner nuclear layer, ONL, outer nuclear layer. Scale bar = 50 μm. (TIF) [file pone.0168651.s001.tif]

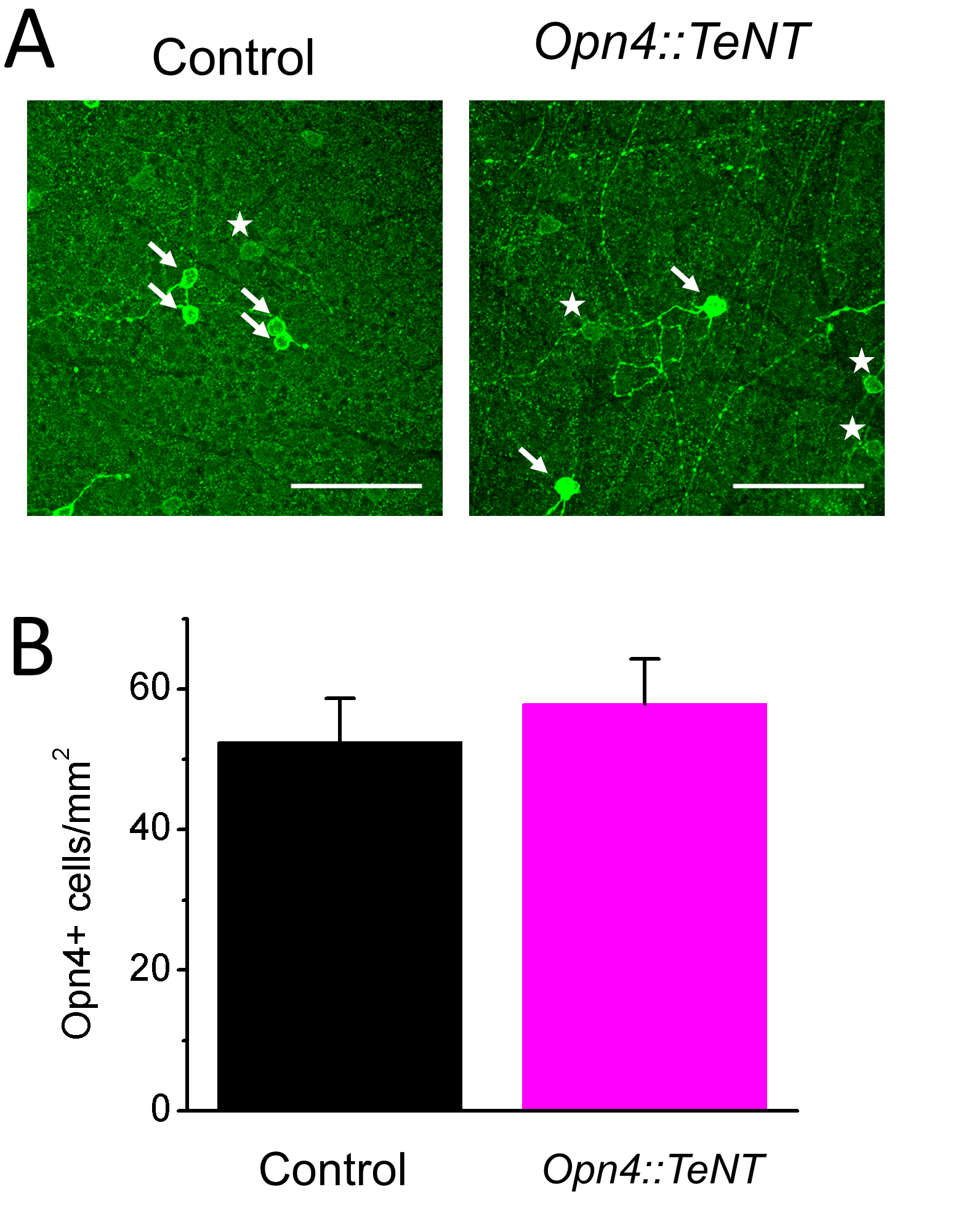

Supplement: S2 Fig — (A) Immunostaining for melanopsin in whole-mount retinas of control and Opn4::TeNT retinas. Confocal images at retinal ganglion cell layer reveals the presence of melanopsin-expressing cells in control and mutant mice retinas. (B) Similar densities of melanopsin expressing cells in the retinal ganglion cell layer of control and Opn4::TeNT retinas (n = 6). Scale bar = 100 μm. (TIF) [file pone.0168651.s002.tif]

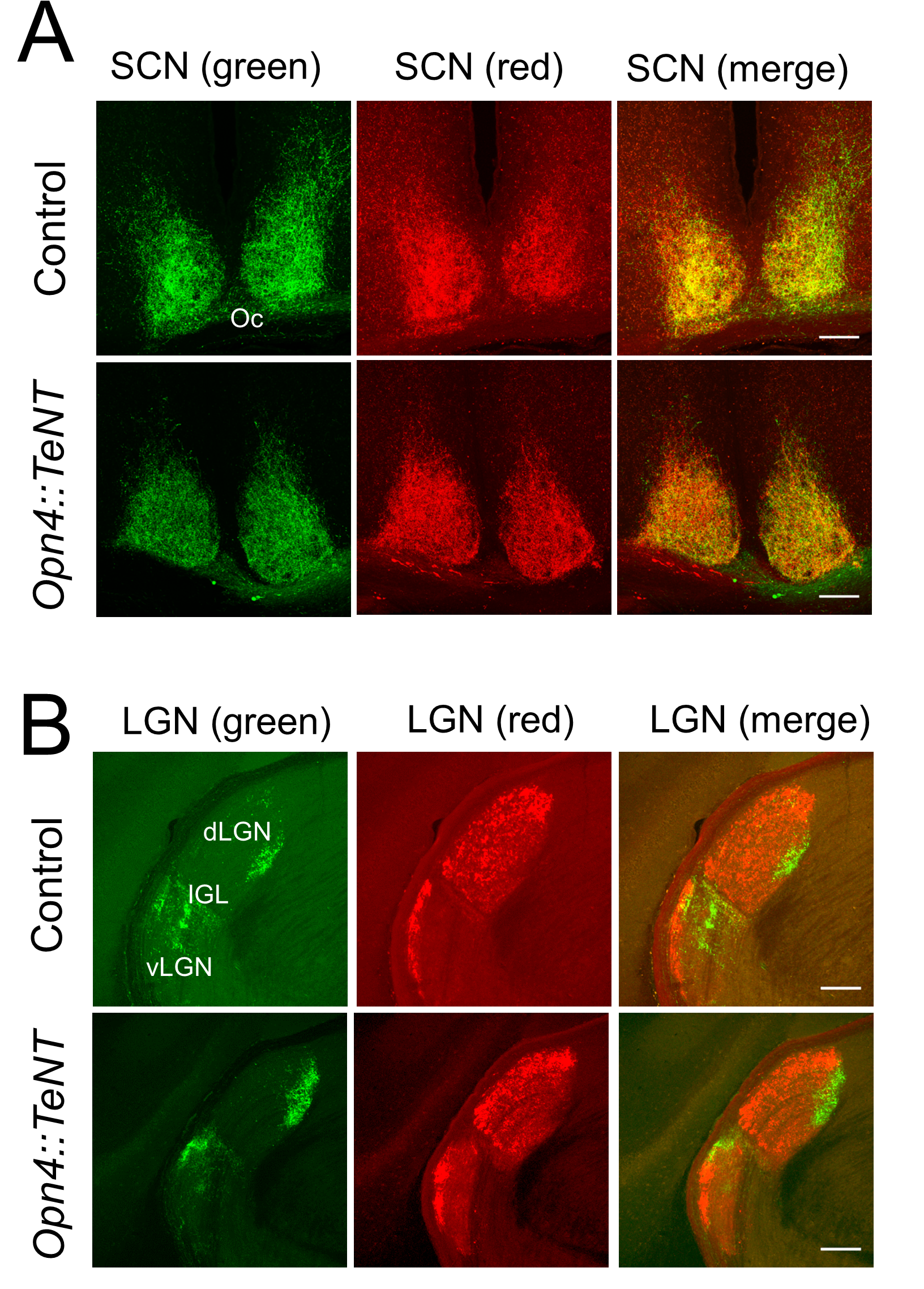

Supplement: S3 Fig — The fidelity of RGC central projections in control and Opn4::TeNT mice adult mice was assessed by anterograde fiber tracing. Mice were killed and perfused, and brain sections analyzed by fluorescence microscopy after injection of Alexa 594–conjugated cholera toxin β into the right eye and Alexa 488–conjugated cholera toxin β into the left eye. Right eye (red) and left eye (green) RGC projections to the SCN (A) and LGN (B). Oc, optic chiasm; dLGN, dorsal LGN; IGL, intergeniculate leafleat; vLGN, ventral LGN. Scale bar = 100 μm for (A) and 200 μm for (B). (TIF) [file pone.0168651.s003.tif]
